# Competition between HIV-1-encoded RRE RNA and miRNA-TRBP interactions alters RNA interference activity and gene expression

**DOI:** 10.1186/1471-2334-14-S2-O13

**Published:** 2014-05-23

**Authors:** A Gatignol, SM Daniels, L Sinck, NJ Ward, CE Melendez-Peña, RJ Scarborough, I Azar, A Daher, KM Pang, JJ Rossi

**Affiliations:** 1 McGill University, Lady Davis Institute for Medical Research, Montreal, Canada; 2 Beckman Research Institute of the City of Hope, Duarte, California, USA

## Aim

RNA interference (RNAi) is a mechanism by which small double-stranded RNAs called micro(mi) or small interfering(si) RNAs bind messenger RNAs (mRNAs) to inhibit their expression. The mechanism involves the RNA-induced silencing complex (RISC) composed of Dicer, TRBP and Ago2 proteins in which TRBP loads miRNAs into the active complex [1]. Several mammalian viruses interfere with RNAi activity. Changes in miRNA and mRNA expression have been observed in patients infected by HIV-1, but the mechanisms are not understood. To explain part of the relationship between RNAi and HIV-1, we investigated the ability and the mechanism of the HIV-1-encoded RNA Rev-Response Element (RRE) to suppress RNAi.

## Methods

We used a model based on miRNA Let7 activity on a reporter gene (RL or EGFP) linked to a complementary sequence (cLet7) to measure RNAi activity or its suppression. We used RNA-immunoprecipitation (IP) and gel mobility shift assays to compare TRBP binding to RRE or siRNAs. We studied RRE activity on RNAi in the context of the entire HIV-1, a lentiviral or an adenoviral vector.

## Results

We observed that RRE, acts as an RNAi suppressor with no modification of the endogenous RISC (Daniels et al., submitted). In contrast, RRE RNA displaces siARNs from TRBP, which suggests a change in miRNA incorporation into the RISC. RNAi remains functional in HIV-1 infected cells, whereas a lentiviral vector expressing RRE has a suppressive activity. The suppression is alleviated when Rev or GagPol is expressed. Adenovirus is known to be suppressed by RNAi and RRE reverses this inhibition as seen by increased viral replication.

## Conclusions

RRE is a new RNAi suppressor, which acts by competition with siRNA and miRNA for binding to TRBP and therefore incorporation into the RISC. This could explain in part the alteration of certain gene expression and modifications of the cell metabolism in patients with long-term HIV-1 infection.
